# Effects of Long COVID in Patients with Severe Coronavirus Disease 2019 on Long-Term Functional Impairments: A Post Hoc Analysis Focusing on Patients Admitted to the ICU in the COVID-19 Recovery Study II

**DOI:** 10.3390/healthcare13040394

**Published:** 2025-02-12

**Authors:** Junji Hatakeyama, Kensuke Nakamura, Shotaro Aso, Akira Kawauchi, Shigeki Fujitani, Taku Oshima, Hideaki Kato, Kohei Ota, Hiroshi Kamijo, Tomohiro Asahi, Yoko Muto, Miyuki Hori, Arisa Iba, Mariko Hosozawa, Hiroyasu Iso

**Affiliations:** 1 Department of Emergency and Critical Care Medicine, Osaka Medical and Pharmaceutical University, 2-7, Daigaku-machi, Takatsuki 569-8686, Osaka, Japan; hatajun1228@yahoo.co.jp; 2 Department of Emergency and Critical Care Medicine, Hitachi General Hospital, 2-1-1 Jonan-cho, Hitachi 317-0077, Ibaraki, Japan; 3 Department of Critical Care Medicine, Yokohama City University Hospital, 3-9 Fukuura, Kanazawa-ku, Yokohama 236-0004, Kanagawa, Japan; 4 Department of Health Services Research, Graduate School of Medicine, The University of Tokyo, 7-3-1 Hongo, Bunkyo-ku 113-0033, Tokyo, Japan; s_aso@m.u-tokyo.ac.jp; 5 Department of Critical Care and Emergency Medicine, Japanese Red Cross Maebashi Hospital, 389-1 Asakura-machi, Maebashi 371-0811, Gunma, Japan; a.kawauchi622@gmail.com; 6 Department of Emergency Medicine and Critical Care Medicine, St. Marianna University, 2-16-1 Sugao, Miyamae-ku, Kawasaki 216-8511, Kanagawa, Japan; shigekifujitani@marianna-u.ac.jp; 7 Department of Emergency and Critical Care Medicine, Chiba University Graduate School of Medicine, 1-8-1 Inohana, Chuo-ku, Chiba 260-8677, Chiba, Japan; t_oshima@chiba-u.jp; 8 Infection Prevention and Control Department, Yokohama City University Hospital, 3-9 Fukuura, Kanazawa-ku, Yokohama 236-0004, Kanagawa, Japan; ekato@yokohama-cu.ac.jp; 9 Department of Emergency and Critical Care Medicine, Hiroshima University Hospital, 1-2-3 Kasumi, Minami-ku, Hiroshima 734-8551, Hiroshima, Japan; kota@hiroshima-u.ac.jp; 10 Department of Emergency and Critical Care Medicine, Shinshu University School of Medicine, 3-1-1 Asahi, Matsumoto 390-8621, Nagano, Japan; hkamijo@shinshu-u.ac.jp; 11 Department of Cardiology, Naha City Hospital, 2-31-1 Furujima, Naha 902-8511, Okinawa, Japan; tasahi@nch.naha.okinawa.jp; 12 Institute for Global Health Policy Research, Bureau of International Health Cooperation, National Center for Global Health and Medicine, 1-21-1 Toyama, Shinjuku-ku 162-8655, Tokyo, Japan; ymutou@it.ncgm.go.jp (Y.M.); miyhori@it.ncgm.go.jp (M.H.); aiba@it.ncgm.go.jp (A.I.); mhosozawa@it.ncgm.go.jp (M.H.); hiso@it.ncgm.go.jp (H.I.)

**Keywords:** COVID-19, functional disability, post-intensive care syndrome, post-COVID-19 condition, long COVID

## Abstract

**Background/Objectives**: This study investigated the prevalence of functional impairments and the effects of long COVID on long-term functional impairments in patients with severe COVID-19. **Methods**: We conducted a nationwide multicenter cohort study in collaboration with nine hospitals, collecting data using self-administered questionnaires from participants aged 20 years or older who were diagnosed with COVID-19, admitted to the intensive care unit (ICU) between April 2021 and September 2021, and discharged alive. Questionnaires regarding daily life, sequela, and functional impairments were mailed to patients in August 2022. The effects of long COVID on functional impairments were examined using a multivariate logistic regression analysis. **Results**: The survey was completed by 220 patients, with a mean of 416 days after discharge. Among respondents, 20.5% had physical impairments (n = 45), 35.0% had mental disorders (n = 77), and 42.7% had either (n = 94). Furthermore, 77.7% had long COVID (171/220), and the most common symptom was dyspnea (40.0%). The multivariate analysis showed that fatigue/malaise, upper respiratory tract symptoms, myalgia, muscle weakness, decreased concentration, sleep disorder, brain fog, and dizziness were risk factors for functional impairments at one year. **Conclusions**: Many patients with severe COVID-19 admitted to the ICU still suffered from post-intensive care syndrome even after one year, which manifested in combination with direct symptoms of the original disease, such as long COVID.

## 1. Introduction

Post-intensive care syndrome (PICS) refers to physical, cognitive, and mental health impairments that occur during an intensive care unit (ICU) stay or after ICU or hospital discharge, and affect the long-term prognosis and quality of life (QOL) of patients who survive ICU discharge [1]. Although various risk factors have been reported for the development of PICS, its prevalence remains high despite preventive measures, and it has become an important social issue due to deteriorating working conditions and induced economic stress [2,3].

The coronavirus disease 2019 (COVID-19) pandemic has had a pronounced impact on human health and daily life, with acute symptoms, such as fatigue/malaise and dyspnea, as well as post-acute symptoms, including hair loss, sleep disorder, and decreased concentration, which are reported as “Long Covid”, “Post COVID-19 Condition”, or “Post Acute Sequelae after severe acute respiratory syndrome coronavirus 2 (SARS-CoV-2) Infection” [4]. They include multifaceted symptoms related to the heart, lungs, gastrointestinal tract, pancreas, immune system, nervous system, blood vessels, kidneys, spleen, liver, and reproductive system [4,5], and may have long-term adverse effects on daily life [6].

PICS and long COVID may overlap in surviving patients with severe COVID-19 who require ICU treatment. PICS is a syndrome that is also affected by symptoms due to the disease itself that caused admission to the ICU [1]. On the other hand, long COVID is clearly defined as sequelae after COVID-19 disease [4]. It remains unclear whether long COVID affects the development of long-term PICS, which is a matter that warrants further investigation. Long COVID, including fatigue, breathing difficulties, and neurological issues, may compound long-term functional impairments and delay recovery. Therefore, we herein investigated the prevalence of long COVID in patients with COVID-19 admitted to the ICU, as well as the prevalence of functional impairments, and then examined the effects of long COVID on long-term functional impairments one year after discharge.

## 2. Methods

### 2.1. Study Design and Setting

The present study was a post hoc analysis of patients admitted to the ICU within COVID-19 Recovery Study II (CORES II). CORES II investigated post-illness symptoms, complications, and physical, mental, and social statuses after COVID-19 by following up with patients hospitalized with COVID-19 after their discharge. The present study included critically ill patients admitted to the ICU in a multicenter study comprising 20 medical institutions, and it was planned in advance to set up an ICU working group (WG) comprising 9 of the 20 medical institutions and to extract the ICU-WG’s own data. This study was approved by the Ethics Committee (Approval No. NCGM-S-004471, Approval Date: 26 April 2022) and was supported by MHLW Research on Emerging and Re-emerging Infectious Diseases and Immunization (Program Grant Number JPMH21HA2011, JPMH23HA2011, JPMH24HA2015). Data in this study were collected and managed using Research Electronic Data Capture, a secure, web-based data capture application hosted at the JCRAC data center of the National Center for Global Health and Medicine [7]. The study complies with the Declaration of Helsinki in its current version. Written informed consent was obtained from all the participants.

### 2.2. Study Population and Eligibility Criteria

Inclusion criteria for CORES II were patients aged 20 years or older who were diagnosed with COVID-19, admitted to the ICU between 1 April 2021 and 30 September 2021, and discharged alive from a cooperating facility. Exclusion criteria for CORES II were patients who were uncontactable (e.g., died after discharge or address unknown), those who were ineligible due to language or cognitive issues, or those who refused prior telephone contact. Patients with pre-existing mental illnesses were excluded from the analysis of mental disorders.

### 2.3. Procedures

Patients who agreed to participate in the present study completed paper-based questionnaires between 1 August 2022 and 30 September 2022 for the survey (approximately one year after discharge). Due to the general lack of established follow-up outpatient clinics after ICU discharge in Japan and the impossibility of frequent direct follow-up of COVID-19 patients, especially during this period of the pandemic, a common mailed questionnaire was used as the method for assessing outcomes in studies evaluating sequelae. Based on the International Severe Acute Respiratory and Emerging Infection Consortium (ISARIC) follow-up questionnaire [8], the presence or absence, as well as the duration, of the following 26 symptoms, were assessed: fever, fatigue/malaise, sore throat, rhinorrhea, cough, dyspnea, chest pain, palpitations, dysgeusia, anosmia, headache, joint pain/joint swelling, myalgia, muscle weakness, anorexia, nausea/vomiting, abdominal pain, sleep disorder, decreased concentration, brain fog, hair loss, skin rash, eye symptoms, dizziness, erectile dysfunction (only for men), and menstrual changes (only for women). For 26 symptoms, we first asked whether symptoms appeared after COVID-19, and if so, “When did they appear?” (from the onset of the disease, or from what year and month), “How long did they last?” (less than 28 days after onset, more than 28 days after onset (until what month of the year), or are they still persistent?). Physical function was examined using the modified Medical Research Council Dyspnea (MRC) scale [9]. The mental health status was measured using the Japanese version of the Hospital Anxiety and Depression Scale (HADS) [10]. Post-traumatic stress disorder (PTSD) symptoms attributable to COVID-19 infection were assessed using a new short version of the Post-traumatic Diagnostic Scale modified for COVID-19 patients, and those who experienced stressful life events related to COVID-19 (i.e., experienced loss due to COVID-19 or were discriminated against due to COVID-19 infections) were also included [11]. The Three-item Loneliness Scale (TIL Scale) was used to assess loneliness [12]. QOL was evaluated before COVID-19 and at the time of the questionnaire response using the self-rated Euro Quality of Life 5 Dimension 5 Level (EQ-5D-5L) [13]. All questionnaire items were listed in a previous study [14].

### 2.4. Variables and Measurements

Patient clinical data are described in previous studies [14], and the following data were also collected: dates of ICU admission and discharge, the sequential organ failure assessment (SOFA) score at ICU admission, delirium and duration of delirium during 1 week of ICU admission, prone position, duration of the continuous administration of a neuromuscular blockade, rehabilitation; duration from ICU admission to the start of the rehabilitation program, ICU mobility scale scores on ICU days 3, 5, and 7, nutritional therapy; calories and protein administered for enteral and parenteral nutrition, respectively, from ICU days 1 to 7, mechanical ventilation (MV); duration of MV, extracorporeal membrane oxygenation (ECMO); duration of ECMO.

### 2.5. Outcomes

The primary outcome was the prevalence of long COVID. Secondary outcomes were the prevalence of physical impairments, mental disorders, and loneliness and the value of EQ-5D-5L in the survey. Long COVID was defined as the continuation or development of new symptoms 3 months after the initial SARS-CoV-2 infection, with these symptoms lasting for at least 2 months with no other explanation according to the WHO definition [15]. Long COVID was diagnosed when the symptoms that patients had matched one or more of the 26 ISARIC symptoms. Physical impairments were defined as a modified MRC score ≥ Grade 2 [9]. Mental disorders were defined as a HADS-Anxiety or HADS-Depression score ≥ 8 or a new short version of the Post-traumatic Diagnostic Scale ≥ 3 [10,11]. Loneliness was defined on the TIL Scale as ≥6 [16].

### 2.6. Statistical Analysis

Continuous variables were presented as medians and interquartile ranges or as means and standard deviations. Categorical variables were presented as absolute values and percentages. The continuous variables of patient characteristics were compared using a Mann–Whitney U test and categorical variables using a chi-squared test. Outcomes were compared with and without ventilatory management to account for the impact of differences in patient severity. To examine and assess risk factors for physical impairments and mental disorders in the respective long COVID symptoms, risk factors were analyzed using a multivariable logistic regression analysis adjusted for age [17], male sex [18], obesity (body mass index ≥ 25.0 kg/m^2^) [19], SOFA at ICU admission [18], delirium [20], and mechanical ventilation [20]. Patient characteristics were selected based on previous studies. In addition, a sensitivity analysis with age [17], male sex [18], obesity (body mass index ≥ 25.0 kg/m^2^) [19], and SOFA at ICU admission [18] as covariates was performed to confirm the robustness of the main results. A *p* value < 0.05 (two-sided) was considered to be significant. All data were examined using STATA SE software, version 17 (Stata Corp, College Station, TX, USA).

## 3. Results

**Figure 1:**
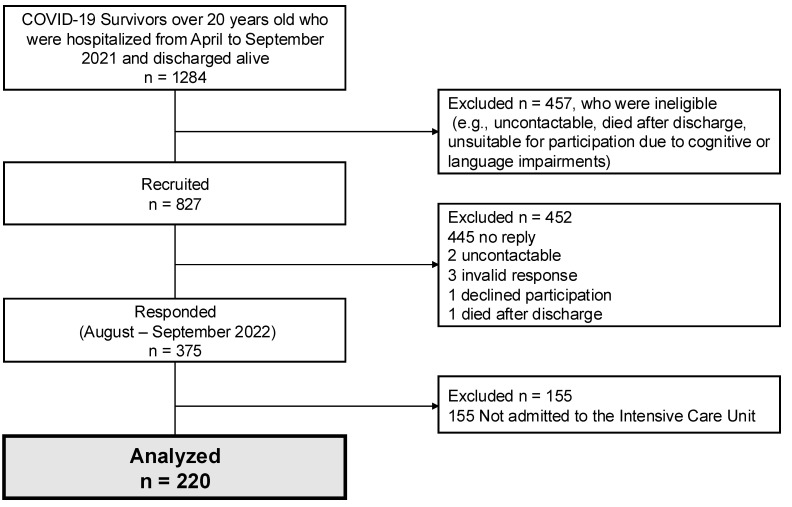
Study outline, flow chart depicting the enrollment of subjects in the present study. COVID-19; coronavirus disease 2019.

The study outline is shown in Figure 1. A total of 220 surviving patients completed the questionnaire in the survey. Patient characteristics in the survey are shown in Table 1. The survey was evaluated with a mean (standard deviation) of 416 (59) days after discharge. Median age was 57 years, the percentage of males was 71.4%, the median body mass index was BMI 27.0 kg/m^2^, the median SOFA score was 5 on ICU admission, and the percentage of patients requiring mechanical ventilation was 71.4%. The percentages of patients requiring ECMO induction, tracheostomy, delirium, neuromuscular blockade, prone position therapy, and rehabilitation were 7.7, 13.6, 34.6, 41.4, 71.4, and 85.0%, respectively. Steroids were administered to the majority of patients. The patient background with and without ventilatory management is shown in Supplemental Table S1. The ventilatory management group had higher SOFA scores and longer hospital stays.

**Figure 2:**
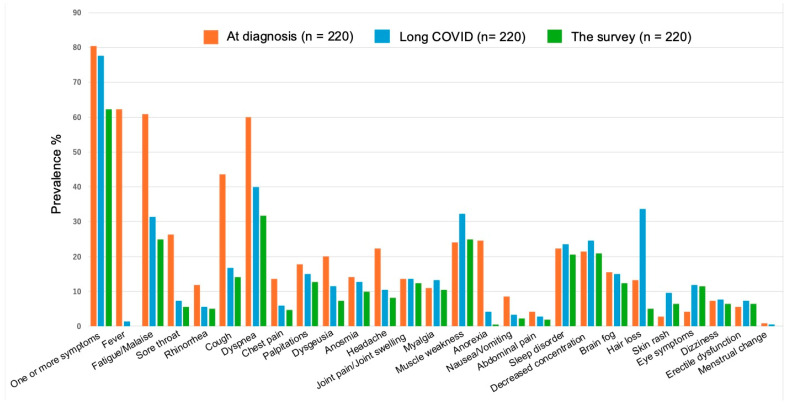
Twenty-six symptom outcomes were selected from the International Severe Acute Respiratory and Emerging Infections Consortium follow-up questionnaires. The prevalence of symptoms of the International Severe Acute Respiratory and Emerging Infections Consortium. The population of each was 220 cases, and the percentages of symptomatic cases are shown in the figure. From left to right, the percentage of patients who were symptomatic at the time of severe acute respiratory syndrome coronavirus 2 (SARS-CoV-2) (orange), the percentage who had the continuation or development of new symptoms 3 months after the initial SARS-CoV-2 infection, with these symptoms lasting for at least 2 months (Long COVID) (light blue), and the percentage who had persistent symptoms until the questionnaire survey (green).

Details on the 26 symptoms in the questionnaire results are shown in Figure 2. The most common symptom for a diagnosis of SARS-CoV-2 infection was fever (62.3%: 137/220), followed by fatigue/malaise (60.9%: 134/220) and dyspnea (60.0%: 131/220). Long COVID was detected in 77.7% (171/220) and the most common symptom was dyspnea (40.0%: 88/220), followed by hair loss (33.6%: 74/220), fatigue/malaise (31.4%: 69/220), and muscle weakness (32.3%: 71/220). Dyspnea (31.8%: 70/220) was the most common symptom that persisted until the time of the survey, followed by fatigue/malaise (25.0%: 44/220), muscle weakness (25.0%: 44/220), decreased concentration (20.9%: 46/220), and sleep disorder (20.5%: 45/220).

The prevalence of physical impairments was 20.5% and that of mental disorders was 35.0% 1 year after infection. Loneliness affected 21.8% of respondents to the survey. The mean value of EQ-5D-5L at the time of the survey was 0.831. Other questionnaire results are shown in Table 2. The percentage of respondents whose living situation was worsened by SARS-CoV-2 infection was 42.3%, and the numbers of respondents taking leave from a job, changing jobs, and resigning from jobs were seven, four, and twelve, respectively, at the time of the survey. The results of the questionnaire with and without ventilatory management are shown in Supplemental Table S2. There was no difference in the prevalence of physical impairments or mental disorders in the ventilatory management group, but there was a higher prevalence of loneliness and a lower QOL.

Table 3 presents the patient background characteristics and ICU treatments stratified by the presence or absence of functional impairments. The prevalence of physical impairments was significantly higher in patients with obesity, higher SOFA scores, prolonged mechanical ventilation, continuous neuromuscular blockade, ECMO, tracheostomy, and lower educational attainment. In contrast, a history of smoking was associated with a higher prevalence of mental disorders.

The results of univariate analyses of long COVID with and without physical impairments and long COVID with and without mental disorders at the time of the survey are shown in Table 4. Persistent symptoms, such as fatigue/malaise, sore throat, cough, dyspnea, muscle weakness, sleep disorder, decreased concentration, and dizziness, were associated with long-term functional impairments. The prevalence of long COVID with and without ventilatory management is shown in Supplemental Table S3. The ventilatory management group had a significantly higher prevalence of long COVID and a higher rate of complaints of rhinorrhea, dyspnea, joint pain and myalgia, muscle weakness, sleep disorder, and brain fog.

**Figure 3:**
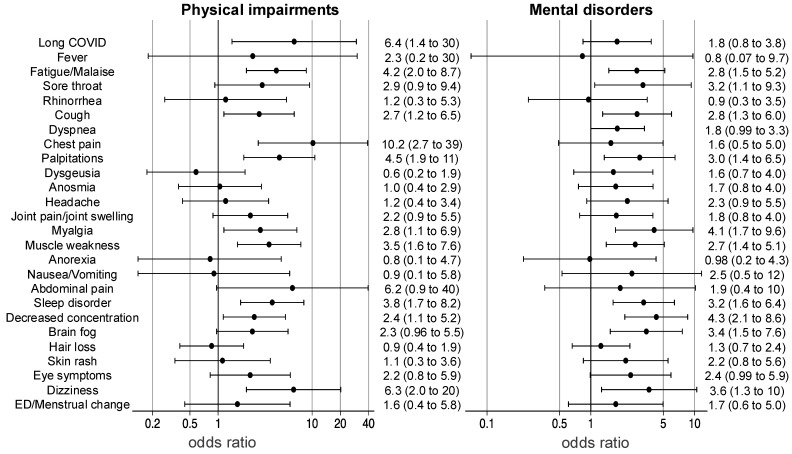
Odds ratios for physical impairments and mental disorders. The results of multiple logistic regression analyses of physical impairments and mental disorders are shown in a forest plot. Functional impairments were adjusted for age, sex, obesity, sequential organ failure assessment scores on intensive care unit admission, delirium, mechanical ventilation, and long COVID symptoms. ED; erectile dysfunction.

The results of the multiple logistic regression analysis of long COVID symptoms in physical impairments and mental disorders are shown in a forest plot (Figure 3). The analysis was adjusted for age, sex, obesity, SOFA on ICU admission, delirium, and mechanical ventilation in the multivariate analysis. Fatigue/malaise (odds ratio [OR] of 4.2, 95% confidence interval [CI] 2.0–8.7), cough (OR 2.7, 95%CI 1.2–6.5), dyspnea (OR 4.4, 95%CI 2.0–9.4), chest pain (OR 10.2, 95%CI 2.7–39), palpitations (OR 4.5, 95%CI 1.9–11), myalgia (OR 2.8, 95%CI 1.1–6.9), muscle weakness (OR 3.5, 95%CI 1.6–7.6), sleep disorder (OR 3.8, 95%CI 1.7–8.2), decreased concentration (OR 2.4, 95%CI 1.1–5.2), and dizziness (OR 6.3, 95%CI 2.0–20) were risk factors for physical impairments at 1 year. Persistent fatigue/malaise (OR 2.8, 95%CI 1.5–5.2), sore throat (OR 3.2, 95%CI 1.1–9.3), cough (OR 2.8, 95%CI 1.3–6.0), palpitations (OR 3.0, 95%CI 1.4–6.5), myalgia (OR 4.1, 95%CI 1.7–9.6), muscle weakness (OR 2.7, 95%CI 1.4–5.1), sleep disorder (OR 3.2, 95%CI 1.6–6.4), decreased concentration (OR 4.3, 95%CI 2.1–8.6), brain fog (OR 3.4, 95%CI 1.5–7.6), and dizziness (OR 3.6, 95%CI 1.3–10) were risk factors for mental disorders at 1 year. Multiple logistic regression analysis of long COVID symptoms in physical impairments and mental disorders with sensitivity analysis using age, sex, obesity, and SOFA on ICU admission as covariates is shown in Supplemental Figure S1. The result in the sensitivity analysis was similar to those in the main analysis.

## 4. Discussion

Among critically ill patients with COVID-19 admitted to the ICU, long COVID was present in 77.7%, with dyspnea, muscle weakness, and fatigue/malaise being the main symptoms persisting in 62.3% for as long as one year. One year after discharge, 42.7% had functional impairments. Risk factors for long-term functional impairments included the long COVID symptoms of fatigue/malaise, upper respiratory symptoms, cardiovascular symptoms, skeletal symptoms, and neurological symptoms. When considering the severity of illness, the prevalence of long COVID was higher in the more severely ill patients who required ventilatory management, but there was no difference in the prevalence of physical impairments and mental disorders.

The most frequent symptoms of long COVID are fatigue/malaise and dyspnea; however, other typical symptoms include headache, myalgia, dysgeusia, anosmia, hair loss, and cardiac and gastrointestinal issues [21]. Long COVID symptoms may persist for 6 months after discharge or even longer, severely affecting a patient’s QOL [22]. SARS-CoV-2 binds to angiotensin-converting enzyme 2 (ACE-2) receptors, which are expressed in many organs, and, thus, potentially affects any system, such as the cardiovascular, central nervous, respiratory, and immune systems; however, its pathophysiology remains unclear [23]. Risk factors for long COVID have also been reported and are related to age, sex, obesity, underlying diseases, and severity [24]. In a meta-analysis of 194 studies, 735,006 participants were included and the prevalence of long COVID was 45% at a mean follow-up of 126 days [25]. Although some symptoms did not appear to be associated with severity [26], many, including fatigue/malaise, were associated with the risk of severity [24]. Long COVID was more common in the present cohort than in previous studies [27]. This may be due to the severity of COVID-19, although various confounding factors and biases in this study may be involved. The prevalence of long COVID was further higher in the severely ill patients who required ventilatory management in this study, which aligns with previous reports showing that the more severe the illness, the higher the prevalence of long COVID [28,29]. It is speculated that patients with severe COVID-19 are more likely to develop long COVID due to direct organ damage from the virus, an excessive inflammatory response and subsequent cytokine storm, and microvascular damage, which may lead to PICS from a pathophysiological perspective [30,31].

The novelty of this study lies in the simultaneous evaluation of long COVID symptoms and PICS, as well as the investigation of their correlation exclusively in patients with severe COVID-19 admitted to the ICU. Although long COVID and PICS are different syndromes, they may overlap in severely ill patients with COVID-19 admitted to the ICU. Long COVID is triggered by SARS-CoV-2 infection and the virus binds to ACE-2 receptors [23]. Some symptoms of PICS also develop from these pathologies [32]; however, PICS may be caused by other factors, including ICU care and its environment, treatments, and drug administration [33]. PICS has a different trajectory depending on functional impairments, and cognitive impairments and mental disorders may not be present on ICU discharge, but may develop later after discharge, and the patient may have functional impairments for a long time afterwards [34,35]. If long COVID symptoms persist for several months, multiple follow-ups may be necessary for functional assessment purposes. While fatigue/malaise is not traditionally regarded as a neuropsychiatric disorder, it may manifest as a symptom of many mental health conditions, such as depression, anxiety, and PTSD [36]. SARS-CoV-2 infiltrates directly into lung tissue, causing a systemic inflammatory response and microvascular damage, which leads to cranial nerve damage and saturates the central nervous system with dysgeusia, anosmia, and visual impairment [37,38]. A meta-analysis reported a relationship between visual impairment and depression [39], and brain fog in long COVID may be a risk factor for mental disorders in the long term. Since the factors of PICS, including mental disorders and cognitive impairments, are related to each other [40,41], sleep disorder and decreased concentration, which are related to cognitive function as long COVID symptoms [42], are also considered to be risk factors for long-term mental disorders. Therefore, long-term mental care and cognitive rehabilitation are needed. The fact that disease-specific sequelae affect PICS reflects the universal nature of PICS, but the heterogeneity of ICU patients has made it difficult to analyze them in the general PICS study. Therefore, this study would possibly suggest future PICS research to reconsider it.

The present study had a significant risk of recall bias because the survey asked about symptoms experienced at the onset of COVID-19, which was approximately one year before the survey was conducted. It may be difficult for patients to accurately remember the presence and timing of symptoms from the acute phase of their illness after such a long interval. This limitation is common among studies investigating the long-term consequences of COVID-19 using questionnaires [43,44]. Recall bias may lead to an underestimation of the prevalence of some symptoms, particularly those that are less severe or persistent [45]. On the other hand, individuals who experienced more severe or prolonged symptoms may be more likely to remember and report them, potentially leading to an overestimation of prevalence [46]. While we cannot determine the direction or magnitude of the impact of recall bias on our results, it is important to interpret our findings with this limitation in mind.

Our study did not allow for a complete cohort analysis that included dropouts, noncritical patients not admitted to the ICU, and non-COVID-19 patients. This issue is not unique to our study and is a common challenge in both PICS and long COVID studies. A systematic review found that the median follow-up rate in PICS studies was only 50%, with a quarter of the studies having a follow-up rate below 30% [47]. Moreover, the review highlighted that the reasons for loss to follow-up were often not reported, making it difficult to assess the potential impact of selection bias [47]. A study that comprehensively evaluated long-term health outcomes for ICU and non-ICU patients admitted with COVID-19 up to two years after discharge showed that most outcomes at two years were comparable between the two groups, although ICU patients tended to have delayed recovery in some areas [48]. This finding underscores the substantial burden of long-term health problems, even among less severely ill COVID-19 patients who did not require ICU admission. It also suggests that factors beyond ICU admission, such as the direct effects of SARS-CoV-2 infection or the inflammatory response, may contribute to the development of persistent symptoms. Similarly, in long COVID studies, a recent meta-analysis highlighted substantial heterogeneity in the study population and the lack of non-COVID controls in many studies [49]. Like ours, these studies provide valuable insights into the long-term outcomes of ICU survivors, but the generalizability of their findings is limited. Patients with serious illness or disability are less likely to respond, potentially leading to an underestimation of the burden of PICS. Conversely, patients who have fully recovered may be less willing to participate, potentially inflating prevalence estimates. Despite these limitations, studies focusing on specific patient subgroups, such as severely ill COVID-19 patients requiring ICU care, offer valuable insights into the long-term consequences of critical illness.

The present results showed poor recovery in physical functioning one year after discharge and the three components of mental disorders (depression, anxiety, and PTSD) were assessed over a one-year period. A previous study reported that fibrotic-like changes persisted as computed tomography findings in patients with COVID-19, even after 2 years, and were more pronounced in patients with more critical illnesses who also had dyspnea [50]. In the present study, physical impairments were defined by a modified MRC score ≥ Grade 2, with residual respiratory symptoms specific to patients with COVID-19 and resulting in poor recovery after one year. Among mental disorders, PTSD rarely occurs in isolation and is often complicated by anxiety and depression [51]. Patients with COVID-19-associated acute respiratory distress syndrome have poor recovery even after 12 months [35], and long-term mental care is required for severely ill patients admitted to the ICU. It may be important to establish a follow-up system that involves follow-up clinics and telemedicine after ICU discharge [52,53]. Follow-up after ICU discharge was found to be useful for some patients, underscoring the need for an individualized approach [54,55]. In addition, because PICS follows a variety of trajectories, it is important to evaluate patients over multiple time points rather than at one single point in time [52,56]. Risk factors identified in our study, such as persistent fatigue/malaise, respiratory symptoms, and neurologic symptoms, can be used to triage patients for more intensive monitoring and targeted interventions.

The present study had several limitations. There was a recall bias because the survey was conducted approximately one year after discharge. Furthermore, there was a survival bias because only surviving patients were included in the survey. There was also a selection bias because those with difficulty completing the questionnaire were excluded. These biases led to lower internal validity and represented significant limitations of this study. Another limitation is that some patients may originally have had a physical impairment. In addition, the self-report assessment methods used in the present study were validated with regards to physical function and mental health; however, only self-reported assessments were conducted. The present study was also based on a mailed questionnaire, and responses to the questionnaire were allowed to be substituted by family members if the patient was unable to respond directly, which may have resulted in better or worse patient ratings by family members. Another limitation is that PICS studies conducted using different assessment tools may not be comparable, and minor physical disabilities may have been missed when using the modified MRC score. Because we were not able to obtain detailed data on patients who were not admitted to the ICU or who were not hospitalized, we were not able to compare patients who were admitted to the ICU with those who were not admitted to the ICU or who were not hospitalized. The risk of recall bias is a common limitation among studies investigating the long-term consequences of COVID-19 using retrospective questionnaires [43,44]. To minimize this bias and provide more reliable estimates of symptom prevalence and trajectories, future studies should employ prospective designs with repeated assessments from the acute phase to long-term follow-up [29]. Such studies would also allow for a more granular analysis of the temporal dynamics of symptom development and persistence, which is critical for understanding the natural history of long COVID [57].

## 5. Conclusions

Long COVID symptoms and PICS were assessed simultaneously and correlated in patients with severe COVID-19 admitted to the ICU: more than 40% (94/220) still had some functional impairment after one year, long COVID was present in more than 70% (171/220), and its sequelae persisted for one year in 60% (137/220). Patients with persistent complaints of fatigue/malaise, upper respiratory tract symptoms, myalgia and muscle weakness, decreased concentration and sleep disorder, brain fog, and dizziness are at risk of long-term functional impairments. While our findings should be interpreted with caution due to the potential for various biases, they highlight the substantial burden of persistent symptoms and functional impairments among ICU survivors of COVID-19 and require comprehensive long-term follow-up care.

## Figures and Tables

**Table 1:** Patient characteristics and clinical course during admission.

	The Survey (n = 220)
Age, years, median (IQR)	57 (50, 61)
Male, n (%)	157 (71.4)
BMI, kg/m^2^, median (IQR)	27.0 (23.4, 30.3)
Obesity (BMI ≥ 25 kg/m^2^), n (%)	144 (65.5)
SOFA at ICU admission, median (IQR)	5 (3, 7)
Clinical frailty scale, median (IQR)	2 (1, 3)
Frailty (clinical frailty scale ≥ 4), n (%)	4 (1.8)
ICU mobility scale, median (IQR)	
day 3	0 (0, 1)
day 5	0 (0, 1)
day 7	0 (0, 3)
Delirium, n (%)	76 (34.6)
Duration of delirium, day, median (IQR)	2 (1, 3)
MV, n (%)	157 (71.4)
Duration of MV, day, median (IQR)	9 (5, 14)
HFNC, n (%)	114 (51.8)
Length of ICU stay, day, median (IQR)	9 (5, 14)
Length of hospital stay, day, median (IQR)	14.5 (8, 26)
Comorbidity, n (%)	132 (60.0)
Hypertension, n (%)	62 (28.2)
Diabetes mellitus, n (%)	58 (26.4)
Heart failure, n (%)	4 (1.8)
COPD, n (%)	10 (4.6)
Cerebrovascular disease, n (%)	10 (4.6)
CKD Grade 5, n (%)	1 (0.5)
Autoimmune disorder, n (%)	7 (3.2)
Immunodeficiency, n (%)	6 (2.7)
Mental disorder, n (%)	10 (4.6)
Malignant tumor, n (%)	18 (8.2)
Prone position, n (%)	157 (71.4)
Continuous neuromuscular blockade, n (%)	91 (41.4)
ECMO, n (%)	17 (7.7)
Duration of ECMO, day, median (IQR)	9 (7, 12)
Rehabilitation, n (%)	187 (85.0)
Time from ICU admission to rehabilitation program initiation, day, median (IQR)	3 (2, 4)
Tracheostomy, n (%)	30 (13.6)
RRT, n (%)	13 (5.9)
Steroid, n (%)	218 (99.1)
Vaccination, n (%)	190 (86.4)
Smoking history, n (%)	138 (62.7)
Population with mandatory 12-year education, n (%)	110 (50.0)

BMI; body mass index, CKD; chronic kidney disease, COPD; chronic obstructive pulmonary disease, ECMO; extracorporeal membrane oxygenation, HFNC; high-flow nasal cannula, ICU; intensive care unit, IQR; interquartile range, MV; mechanical ventilation, RRT; renal replacement therapy, SOFA; sequential organ failure assessment.

**Table 2:** Questionnaire results one year after discharge.

	The Survey (n = 220)
Physical impairments, n (%)	45 (20.5)
Mental disorders, n (%)	77 (35.0)
Anxiety	43 (20.0)
Depression	57 (25.9)
PTSD	33 (15.0)
Physical impairments or mental disorders, n (%)	94 (42.7)
Physical impairments and mental disorders, n (%)	23 (10.5)
HADS, mean (SD)	10.3 (7.8)
HADS-Anxiety, mean (SD)	4.9 (3.8)
HADS-Depression, mean (SD)	5.2 (4.6)
PTSD score, mean (SD)	1.1 (1.6)
Loneliness, n (%)	48 (21.8)
Three-item loneliness scale, mean (SD)	3.1 (2.4)
EQ-5D-5L, mean (SD)	0.831 (0.171)
Unfair discrimination due to SARS-CoV-2 infection, n (%)	37 (16.8)
Living conditions have deteriorated, n (%)	93 (42.3)
Changes in the workplace, n (%)	48 (21.8)
Changes in employment, n (%)	33 (15)
Temporary retirement, n (%)	8 (3.6)
Health deterioration due to SARS-CoV-2 infection, n (%)	7 (3.2)
Change in occupation, n (%)	4 (1.8)
Health deterioration due to SARS-CoV-2 infection, n (%)	4 (1.8)
Retirement, n (%)	16 (7.3)
Health deterioration due to SARS-CoV-2 infection, n (%)	12 (5.5)

EQ-5D-5L; Euro Quality of Life 5 Dimension 5 Level, HADS; Hospital Anxiety and Depression Scale, PTSD; post-traumatic stress disorder, SARS-CoV-2; severe acute respiratory syndrome coronavirus 2, SD; standard deviation.

**Table 3:** Patient characteristics and clinical course by functional impairments.

	Physical Impairments (n = 45)	Non-Physical Impairments (n = 172)	*p* Value	Mental Disorders (n = 77)	Non-Mental Disorders (n = 133)	*p* Value
Age, yr, median (IQR)	57 (50, 63)	56 (50, 61)	0.41	56 (51, 60)	57 (49, 62)	0.58
Male, n (%)	28 (62.2)	127 (73.8)	0.13	56 (72.7)	95 (71.4)	0.84
BMI, kg/m^2^, median (IQR)	27.6 (25.8, 33.1)	26.7 (23.2, 30.0)	0.03	27.0 (23.5, 30.6)	26.9 (23.4, 29.9)	0.51
Obesity (BMI ≥ 25 kg/m^2^), n (%)	36 (81.8)	107 (62.2)	0.01	52 (67.5)	86 (65.2)	0.73
SOFA at ICU admission, median (IQR)	6 (4, 8)	4 (3, 7)	0.02	4 (3, 7)	5 (3, 7)	0.95
Clinical frailty scale, median (IQR)	2 (1, 3)	2 (1, 3)	0.76	2 (1, 3)	2 (1, 3)	0.73
Frailty (CSF ≥ 4), n (%)	1 (2.7)	3 (2.2)	0.85	2 (3.0)	2 (2.0)	0.68
ICU mobility scale, median (IQR)						
day 3	0 (0, 0)	0 (0, 1)	0.26	0 (0, 1)	0 (0, 1)	0.95
day 5	0 (0, 1)	0 (0, 1)	0.41	0 (0, 3)	0 (0, 1)	0.37
day 7	0 (0, 3)	0 (0, 3)	0.99	0 (0, 3)	0 (0, 3)	0.86
Delirium, n (%)	18 (40.0)	58 (33.7)	0.43	29 (37.7)	45 (33.8)	0.58
Duration of delirium, day, median (IQR)	2 (1, 3)	2 (1, 3)	0.49	2 (1, 2)	2 (1, 4)	0.15
MV, n (%)	34 (75.6)	123 (71.5)	0.59	54 (70.1)	95 (71.4)	0.84
Duration of MV, day, median (IQR)	13 (6, 26)	8 (5, 12)	0.006	7.5 (5, 14)	9 (5, 13)	0.85
HFNC, n (%)	22 (48.9)	89 (51.7)	0.73	41 (53.3)	70 (52.6)	0.93
Length of ICU stay, day, median (IQR)	11 (7, 26)	8.5 (5, 13)	0.01	9 (6, 14)	8 (5, 14)	0.19
Length of hospital stay, day, median (IQR)	20 (11, 40)	14 (8, 23.5)	0.008	14 (9, 25)	15 (8, 24)	0.65
Comorbidity, n (%)	26 (57.8)	103 (59.9)	0.80	48 (62.3)	77 (57.9)	0.53
Hypertension	9 (20.0)	52 (30.2)	0.17	26 (33.8)	33 (24.8)	0.16
Diabetes mellitus	12 (26.7)	44 (25.6)	0.88	18 (23.4)	37 (27.8)	0.48
Heart failure	1 (2.2)	3 (1.7)	0.83	2 (2.6)	2 (1.5)	0.58
COPD	2 (4.4)	7 (4.1)	0.91	5 (6.5)	4 (3.0)	0.23
Cerebrovascular disease	1 (2.2)	8 (4.7)	0.47	2 (2.6)	7 (5.3)	0.36
CKD Grade 5	1 (2.2)	0 (0)	0.05	1 (1.3)	0 (0)	0.19
Autoimmune disorder	1 (2.2)	6 (3.5)	0.67	2 (2.6)	3 (2.3)	0.88
Immunodeficiency	2 (4.4)	4 (2.3)	0.44	1 (1.3)	4 (3.0)	0.43
Mental disorder	5 (11.1)	5 (2.9)	0.02	0 (0)	9 (6.8)	0.02
Malignant tumor	2 (4.4)	16 (9.3)	0.29	4 (5.2)	13 (9.8)	0.24
Prone position, n (%)	37 (82.2)	118 (68.6)	0.07	56 (72.7)	94 (70.7)	0.75
Continuous neuromuscular blockade, n (%)	26 (57.8)	65 (37.8)	0.02	37 (48.1)	46 (34.6)	0.05
ECMO, n (%)	7 (15.6)	10 (5.8)	0.03	5 (6.5)	12 (9.0)	0.52
Duration of ECMO, day, median (IQR)	8 (7, 17)	9.5 (7, 12)	0.76	17 (9, 21)	8 (6.5, 10.5)	0.10
Rehabilitation, n (%)	37 (82.2)	147 (85.5)	0.59	64 (83.1)	115 (86.5)	0.51
Time from ICU admission to rehabilitation program initiation, day, median (IQR)	3 (2, 6)	3 (2, 4)	0.25	3 (2, 5)	3 (2, 4)	0.19
Tracheostomy, n (%)	16 (35.6)	14 (8.1)	<0.01	11 (14.3)	17 (12.8)	0.76
RRT, n (%)	5 (11.1)	7 (4.1)	0.07	5 (6.5)	8 (6.0)	0.89
Steroid, n (%)	45 (100)	170 (98.8)	0.47	76 (98.7)	132 (99.3)	0.69
Vaccination, n (%)	41 (91.1)	146 (85.4)	0.32	66 (85.7)	115 (86.5)	0.88
Smoking history, n (%)	28 (62.2)	108 (63.5)	0.87	56 (72.7)	75 (56.8)	0.02
Population with mandatory 12-year education, n (%)	32 (71.1)	75 (44.4)	0.001	42 (55.3)	60 (45.5)	0.17
From discharge to completion of the survey, day, mean (SD)	412 (62)	418 (58)	0.51	417 (60)	415 (58)	0.83

BMI; body mass index, CKD; chronic kidney disease, COPD; chronic obstructive pulmonary disease, CSF; clinical frailty scale, ECMO; extracorporeal membrane oxygenation, HFNC; high-flow nasal cannula, ICU; intensive care unit, IQR; interquartile range, MV; mechanical ventilation, RRT; renal replacement therapy, SD; standard deviation, SOFA; sequential organ failure assessment.

**Table 4:** Prevalence of long COVID symptoms according to the presence or absence of functional impairments one year after discharge.

Long COVID Symptoms, n (%)	Physical Impairments (n = 45)	Non-Physical Impairments (n = 172)	*p* Value	Mental Disorders (n = 77)	Non-Mental Disorders (n = 133)	*p* Value
Long COVID (one or more symptoms)	43 (95.6)	127 (73.8)	0.002	64 (83.1)	99 (74.4)	0.15
Fever	1 (2.2)	2 (1.2)	0.59	1 (1.3)	2 (1.5)	0.90
Fatigue/malaise	26 (57.8)	42 (24.4)	<0.001	35 (45.5)	33 (24.8)	0.002
Sore throat	6 (13.3)	10 (5.8)	0.09	10 (13.0)	6 (4.5)	0.03
Rhinorrhea	3 (6.7)	9 (5.2)	0.71	4 (5.2)	7 (5.3)	0.98
Cough	13 (28.9)	24 (14.0)	0.02	20 (26.0)	15 (11.3)	0.006
Dyspnea	31 (68.9)	57 (33.1)	<0.001	38 (49.4)	47 (35.3)	0.046
Chest pain	9 (20.0)	4 (2.3)	<0.001	6 (7.8)	7 (5.3)	0.46
Palpitations	15 (33.3)	18 (10.5)	<0.001	19 (24.7)	14 (10.5)	0.007
Dysgeusia	4 (8.9)	21 (12.2)	0.54	11 (14.3)	12 (9.0)	0.24
Anosmia	6 (13.3)	22 (12.8)	0.92	13 (16.9)	14 (10.5)	0.19
Headache	6 (13.3)	17 (9.9)	0.50	12 (15.6)	10 (7.5)	0.07
Joint pain/joint swelling	10 (22.2)	20 (11.6)	0.07	14 (18.2)	15 (11.3)	0.16
Myalgia	12 (26.7)	17 (9.9)	0.003	18 (23.4)	10 (7.5)	0.001
Muscle weakness	24 (53.3)	47 (27.3)	0.001	34 (44.2)	33 (24.8)	0.004
Anorexia	2 (4.4)	6 (3.5)	0.76	3 (3.9)	6 (4.5)	0.83
Nausea/vomiting	2 (4.4)	5 (2.9)	0.60	4 (5.2)	3 (2.3)	0.25
Abdominal pain	4 (8.9)	2 (1.2)	0.005	3 (3.9)	3 (2.3)	0.49
Sleep disorder	20 (44.4)	32 (18.6)	<0.001	29 (37.7)	22 (16.5)	0.001
Decreased concentration	19 (42.2)	34 (19.8)	0.002	31 (40.3)	22 (16.5)	<0.001
Brain fog	12 (26.7)	21 (12.2)	0.02	20 (26.0)	13 (9.8)	0.002
Hair loss	16 (35.6)	57 (33.1)	0.76	28 (36.4)	43 (32.3)	0.55
Skin rash	5 (11.1)	16 (9.3)	0.72	11 (14.3)	10 (7.5)	0.12
Eye symptoms	9 (20.0)	16 (9.3)	0.045	13 (16.9)	12 (9.0)	0.09
Dizziness	9 (20.0)	8 (4.7)	0.001	11 (14.3)	6 (4.5)	0.01
Erectile dysfunction/menstrual change	4 (8.9)	13 (7.6)	0.77	8 (10.4)	8 (6.0)	0.25

Long COVID was diagnosed when the symptoms that patients had matched 1 or more of the 26 ISARIC symptoms.

## Data Availability

The data that support the findings of this study are available on reasonable request to the CORESII Research office (cores2@it.ncgm.go.jp).

## Supplementary Materials

The following supporting information can be downloaded at: https://www.mdpi.com/article/10.3390/healthcare13040394/s1.
