# Supplementary material for: Effects of Long COVID in Patients with Severe Coronavirus Disease 2019 on Long-Term Functional Impairments: A Post Hoc Analysis Focusing on Patients Admitted to the ICU in the COVID-19 Recovery Study II
Source: Healthcare (Basel). 2025 Feb 12;13(4):394. doi: 10.3390/healthcare13040394 (PMC11855593; doi:10.3390/healthcare13040394)

Supplemental Material

Effects of Long COVID in Patients with Severe Coronavirus Disease 2019 on Long-Term Functional Impairments: A Post Hoc Analysis Focusing on Patients Admitted to the ICU in the COVID-19 Recovery Study II

Supplemental Table S1. Patient background with and without ventilatory management

Supplemental Table S2. Questionnaire results one year after discharge with and without ventilatory management

Supplemental Table S3. Prevalence of Long COVID symptoms with and without ventilatory management

Supplemental Figure S1. Odds Ratios in Sensitivity Analysis of Physical impairments and Mental Disorders

Supplemental Table S1. Patient characteristics and clinical course during admission with and without ventilatory management

	Ventilatory Management (n = 157)	Non-Ventilatory Management (n = 63)	p value
Age, years, median (IQR)	57 (50, 61)	55 (47, 61)	0.18
Male, n (%)	115 (73.3)	42 (66.7)	0.33
BMI, kg/m^2, median (IQR)	27.0 (24.5, 30.3)	26.4 (22.8, 30.6)	0.52
Obesity (BMI ≥25 kg/m^2), n (%)	109 (69.4)	35 (55.6)	0.07
SOFA at ICU admission, median (IQR)	5 (4, 8)	3 (2, 4)	<0.001
Clinical frailty scale, median (IQR)	2 (1, 3)	2 (2, 3)	0.04
Frailty (clinical frailty scale ≥4), n (%)	2 (1.5)	2 (4.7)	0.23
ICU mobility scale, median (IQR)			
day 3	0 (0, 0)	1 (0, 3)	<0.001
day 5	0 (0, 1)	3 (1, 6)	<0.001
day 7	0 (0, 1)	3 (1, 8)	<0.001
Delirium, n (%)	73 (46.5)	3 (4.8)	<0.001
Duration of delirium, day, median (IQR)	2 (1, 3)	2 (1, 3)	0.91
HFNC, n (%)	64 (40.8)	50 (79.4)	<0.001
Length of ICU stay, day, median (IQR)	11 (7, 15)	5 (3, 7)	<0.001
Length of hospital stay, day, median (IQR)	18 (9, 28)	11 (6, 17)	0.0001
Comorbidity, n (%)	97 (61.8)	35 (55.6)	0.39
Hypertension, n (%)	48 (30.6)	14 (22.2)	0.21
Diabetes mellitus, n (%)	42 (26.8)	16 (25.4)	0.84
Heart failure, n (%)	2 (1.3)	2 (3.2)	0.34
COPD, n (%)	5 (3.2)	5 (7.9)	0.13
Cerebrovascular disease, n (%)	5 (3.2)	5 (7.9)	0.13
CKD Grade 5, n (%)	0 (0)	1 (1.6)	0.11
Autoimmune disorder, n (%)	6 (3.8)	1 (1.6)	0.39
Immunodeficiency, n (%)	6 (3.8)	0 (0)	0.11
Mental disorder, n (%)	9 (5.7)	1 (1.6)	0.18
Malignant tumor, n (%)	17 (10.8)	1 (1.6)	0.02
Prone position, n (%)	118 (75.2)	39 (61.9)	0.049
Rehabilitation, n (%)	144 (91.7)	43 (68.3)	<0.001
Time from ICU admission to rehabilitation program initiation, day, median (IQR)	3 (2, 4)	3 (2, 4)	0.32
RRT, n (%)	9 (5.7)	4 (6.4)	0.86

Steroid, n (%)	155 (98.7)	63 (100)	0.37
Vaccination, n (%)	141 (89.8)	49 (79.0)	0.03
Smoking history, n (%)	101 (64.3)	37 (60.7)	0.61
Population with mandatory 12-year education, n (%)	76 (49.0)	34 (54.8)	0.44

BMI; body mass index, CKD; chronic kidney disease, COPD; chronic obstructive pulmonary disease, HFNC; high-flow nasal cannula, ICU; intensive care unit, IQR; interquartile range, RRT; Renal replacement therapy, SOFA; sequential organ failure assessment

Supplemental Table S2. Questionnaire results one year after discharge with and without ventilatory management

	Ventilatory Management (n = 157)	Non-Ventilatory Management (n = 63)	p value
Physical impairments, n (%)	34 (21.7)	11 (18.3)	0.59
Mental disorders, n (%)	54 (36.2)	23 (37.7)	0.84
Anxiety	30 (20.0)	13 (21.3)	0.83
Depression	40 (25.8)	17 (27.4)	0.81
PTSD	22 (14.0)	11 (17.7)	0.49
Physical impairments or mental disorders, n (%)	68 (45.6)	26 (44.8)	0.92
Physical impairments and mental disorders, n (%)	18 (11.6)	5 (8.3)	0.49
HADS, mean (SD)	10.7 (8.0)	9.3 (7.2)	0.27
HADS-Anxiety, mean (SD)	5.1 (3.7)	4.4 (3.9)	0.15
HADS-Depression, mean (SD)	5.4 (4.9)	4.9 (3.9)	0.87
PTSD score, mean (SD)	1.2 (1.5)	1.0 (1.7)	0.27
Loneliness, n (%)	40 (25.5)	8 (12.9)	0.04
Three-Item loneliness scale, mean (SD)	3.2 (2.6)	2.9 (2.1)	0.45
EQ-5D-5L, mean (SD)	0.812 (0.175)	0.878 (0.150)	0.008
Unfair discrimination due to SARS-CoV-2 infection, n (%)	28 (17.8)	9 (14.3)	0.53
Living conditions have deteriorated, n (%)	70 (44.6)	23 (36.5)	0.27
Changes in the workplace, n (%)	33 (21.3)	15 (24.6)	0.60
Changes in employment, n (%)	27 (17.5)	6 (10.0)	0.17
Temporary retirement, n (%)	7 (4.5)	1 (1.6)	0.30
Health deterioration due to SARS-CoV-2 infection, n (%)	6 (3.8)	1 (1.6)	0.39
Change of occupation, n (%)	2 (1.3)	2 (3.2)	0.34
Health deterioration due to SARS-CoV-2 infection, n (%)	2 (1.3)	2 (3.2)	0.34
Retirement, n (%)	11 (7.0)	5 (7.9)	0.81
Health deterioration due to SARS-CoV-2 infection, n (%)	9 (5.7)	3 (4.8)	0.77

EQ-5D-5L; Euro Quality of Life 5 Dimension 5 Level, HADS; Hospital Anxiety and Depression Scale, PTSD; post-traumatic stress disorder, SARS-CoV-2; severe acute respiratory syndrome coronavirus 2, SD; standard deviation

Supplemental Table S3. Prevalence of Long COVID symptoms with and without ventilatory management

Long COVID symptoms, n (%)	Ventilatory Management (n = 157)	Non-Ventilatory Management (n = 63)	p value
Long COVID (one or more symptoms)	131 (83.4)	40 (63.5)	0.001
Fever	3 (1.9)	0 (0)	0.27
Fatigue/malaise	51 (32.5)	18 (28.6)	0.57
Sore throat	14 (8.9)	2 (3.2)	0.14
Rhinorrhea	12 (7.6)	0 (0)	0.02
Cough	31 (19.8)	6 (9.5)	0.07
Dyspnea	71 (45.2)	17 (27.0)	0.01
Chest pain	10 (6.4)	3 (4.8)	0.65
Palpitations	25 (15.9)	8 (12.7)	0.55
Dysgeusia	18 (11.5)	7 (11.1)	0.94
Anosmia	22 (14.0)	6 (9.5)	0.37
Headache	18 (11.5)	5 (7.9)	0.44
Joint pain/joint swelling	27 (17.2)	3 (4.8)	0.02
Myalgia	26 (16.6)	3 (4.8)	0.02
Muscle weakness	64 (40.8)	7 (11.1)	<0.001
Anorexia	6 (3.8)	3 (4.8)	0.75
Nausea/vomiting	5 (3.2)	2 (3.2)	0.99
Abdominal pain	6 (3.8)	0 (0)	0.12
Sleep disorder	43 (27.4)	9 (14.3)	0.04
Decreased concentration	43 (27.4)	11 (17.5)	0.12
Brain fog	29 (18.5)	4 (6.4)	0.02
Hair loss	57 (36.3)	17 (27.0)	0.19
Skin rash	14 (8.9)	7 (11.1)	0.62
Eye symptoms	20 (12.7)	6 (9.5)	0.50
Dizziness	12 (7.6)	5 (7.9)	0.94
Erectile dysfunction/menstrual change	11 (7.0)	6 (9.5)	0.53

Long COVID was diagnosed when the symptoms that patients had matched one or more of the 26 ISARIC symptoms.

Supplemental Figure S1. Odds Ratios in Sensitivity Analysis of Physical impairments and Mental Disorders

The results of the sensitivity analysis of multiple logistic regression analysis of physical impairments and mental disorders are shown in a forest plot. Functional impairments were adjusted for age, sex, obesity, sequential organ failure assessment score on intensive care unit admission, and Long COVID symptoms. ED, erectile dysfunction

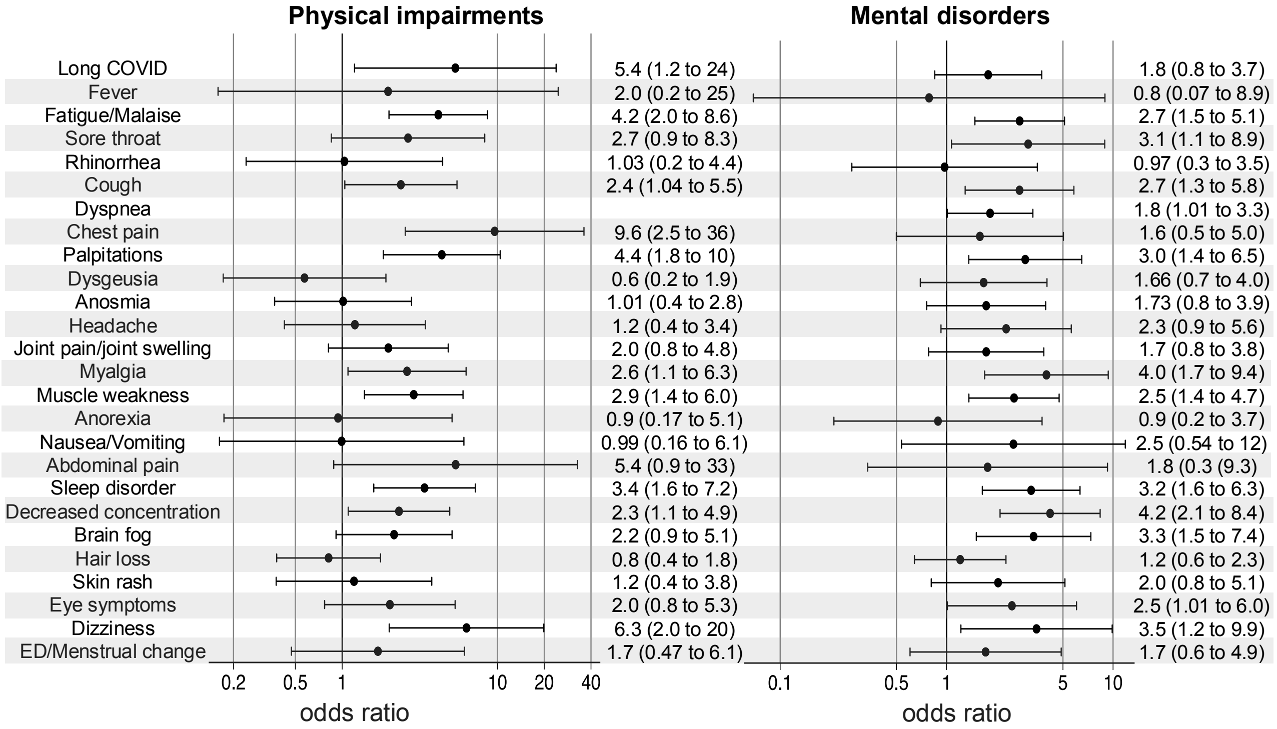

Supplement: Supplementary file 1 [file healthcare-13-00394-s001.zip › healthcare-3397427-supplementary.pdf]
